# Feeding Kinematics, Suction, and Hydraulic Jetting Performance of Harbor Seals (*Phoca vitulina*)

**DOI:** 10.1371/journal.pone.0086710

**Published:** 2014-01-24

**Authors:** Christopher D. Marshall, Sven Wieskotten, Wolf Hanke, Frederike D. Hanke, Alyssa Marsh, Brian Kot, Guido Dehnhardt

**Affiliations:** 1 Department of Marine Biology, Texas A&M University, Galveston, Texas, United States of America; 2 Department of Wildlife and Fisheries Sciences, Texas A&M University, College Station, Texas, United States of America; 3 Institute for Biosciences, Sensory and Cognitive Ecology Group, University of Rostock, Rostock, Germany; Universität Bielefeld, Germany

## Abstract

The feeding kinematics, suction and hydraulic jetting capabilities of captive harbor seals (*Phoca vitulina*) were characterized during controlled feeding trials. Feeding trials were conducted using a feeding apparatus that allowed a choice between biting and suction, but also presented food that could be ingested only by suction. Subambient pressure exerted during suction feeding behaviors was directly measured using pressure transducers. The mean feeding cycle duration for suction-feeding events was significantly shorter (0.15±0.09 s; P<0.01) than biting feeding events (0.18±0.08 s). Subjects feeding in-water used both a suction and a biting feeding mode. Suction was the favored feeding mode (84% of all feeding events) compared to biting, but biting comprised 16% of feeding events. In addition, seals occasionally alternated suction with hydraulic jetting, or used hydraulic jetting independently, to remove fish from the apparatus. Suction and biting feeding modes were kinematically distinct regardless of feeding location (in-water vs. on-land). Suction was characterized by a significantly smaller gape (1.3±0.23 cm; P<0.001) and gape angle (12.9±2.02°), pursing of the rostral lips to form a circular aperture, and pursing of the lateral lips to occlude lateral gape. Biting was characterized by a large gape (3.63±0.21 cm) and gape angle (28.8±1.80°; P<0.001) and lip curling to expose teeth. The maximum subambient pressure recorded was 48.8 kPa. In addition, harbor seals were able to jet water at food items using suprambient pressure, also known as hydraulic jetting. The maximum hydraulic jetting force recorded was 53.9 kPa. Suction and hydraulic jetting where employed 90.5% and 9.5%, respectively, during underwater feeding events. Harbor seals displayed a wide repertoire of behaviorally flexible feeding strategies to ingest fish from the feeding apparatus. Such flexibility of feeding strategies and biomechanics likely forms the basis of their opportunistic, generalized feeding ecology and concomitant breadth of diet.

## Introduction

Secondarily adapted aquatic tetrapods (e.g., marine turtles, penguins, and marine mammals) represent an evolutionarily interesting experiment in organismal adaptation. The aquatic environment has imposed strong selection pressures on all aquatic vertebrates, particularly for feeding and locomotion. This has also been true for terrestrial vertebrates re-invading aquatic habitats. Raptorial feeding is the ancestral condition among all gnathostomes, but the evolution of suction feeding is an especially important feeding mode among actinopterygians, as well as chondrichthyans [1]–[5]. The terrestrial ancestors of marine mammals are thought to have exhibited a raptorial-type feeding mode [6]. Therefore, part of their transition back to the sea likely involved an independent and secondary emergence of new mechanisms for underwater feeding. Our knowledge regarding these adaptations to underwater feeding and the feeding repertoire of aquatic mammals is limited to only a few species and much work needs to be conducted to understand feeding adaptations of marine mammals from a comparative perspective. For example, it is not well known how widespread suction feeding is among aquatic mammals, and in the case of pinnipeds, a generalist (pierce) biting feeding mode is often evoked to describe their ancestral feeding mode [6]. The lack of feeding performance data for secondarily aquatic tetrapods is due to the fact that they spend considerable time at depth foraging and direct observation of prey capture is rare.

The field of feeding functional morphology among secondarily aquatic tetrapods is still in its infancy but has benefitted greatly from investigations of fish and amphibian feeding biomechanics [4], [7]–[11]. Among secondarily aquatic tetrapods suction feeding specialists, such as walruses (*Odobenus rosmarus*), bearded seals (*Erignathus barbatus*), pygmy sperm whales *(Kogia* sp) and belugas (*Delphinapterus leucas*), the primary mechanism of generating subambient pressure is the rapid depression and retraction of the tongue and hyoid apparatus [12]–[18]. This rapid depression of the tongue and the hyoid apparatus can be observed externally (gular depression) in feeding pinnipeds and cetaceans [15], [17]–[19]. The rapid change in intraoral volume generates significant subambient pressure. In these specialists, the tongue is wide, thick and piston-like [13], [14], [16]. It has been suggested that suction specialists also possess an enlarged hyoid apparatus [20]–[22], which presumes a hypertrophied hyolingual musculature and more forceful contraction. While this may be true for some taxa, it does not hold for all suction feeders. For example, Bloodworth and Marshall [16] compared the hyolingual musculature of pygmy and dwarf sperm whales (suction specialists) and bottlenose dolphins (a ram feeder), as well as the feeding kinematics in these species [19], and found no evidence of increase muscle tension capability in suction specialists. Instead modified orofacial morphology is thought to be of greater importance [16], as well as shape of the mandible and head [23]–[25]. Shape of the mouth and the occlusion of lateral gape are important for generating and orienting suction forces for prey capture [5]. Walruses and bearded seals have broad skulls [26], [27] and muscular snouts [28], [29] which they use to purse and create a circular aperture rostrally, and occlude lateral gape by pursing the lateral margins of the lips and mouth. Salamanders occlude lateral gape using labial lobes [10], [30], [31], actinopterygian fish use membranous labial lips that span the upper and lower jaws [32] and suction feeding elasmobranchs occlude lateral gape using labial cartilages [4], [33], [34]. In addition, the vaulted upper palate of suction feeding specialists is hypothesized to increase the rapid volume change, when the tongue is rapidly depressed, to further maximize subambient pressure generation [13], [26]. Rapid lower jaw opening, while maintaining small gape, can also contribute to subambient pressure generation in some species (bearded seals) [17] due to buccal expansion as long as a circular aperture is maintained and the lips occlude the lateral sides of the mouth.

Understanding prey-capture tactics and feeding performance are important considerations in trophic ecological questions since such behavior can determine prey choice due to energetic constraints [35]–[37]. The primary methods of studying pinniped foraging ecology have been through indirect methods (e.g., stomach content and fecal analyses), and more recently through the use of animal-born cameras [37]–[44] and other instrumentation. While such underwater footage can reveal much regarding hunting tactics, the actual moment of prey capture is often obscured and performance measurement of prey capture in the wild is limited. Although the marine mammal literature is rich in correlations among anatomy and feeding modes, studies that have collected direct empirical data of marine mammal feeding performance are limited. Among odontocetes, only belugas (*Delphinapterus leucas*), pygmy and dwarf sperm whales (*Kogia breviceps, K. sima*) and longfinned pilot whales (*Globicephala melas*) have been demonstrated to use suction as a primary feeding mode, whereas only bottlenose dolphins (*Tursiops truncatus*), and Pacific white-sided dolphins (*Lagenorhynchus obliquidens*) have been shown to primarily use a ram or raptorial feeding mode [18], [19], although this is likely a common feeding mode among many odontocetes. Sirenian feeding biomechanics and food handling have received some attention; their feeding involves the use of modified vibrissae, or bristles, for gathering vegetation and transporting it to the cheek-teeth for mastication, but no evidence for suction has been reported [45]–[47]. Data for pinnipeds are also few, but a mix of kinematic and performance data exists for bearded seals (*Erignathus barbatus*) [17], leopard seals (*Hydrurga leptonyx*) [48], and walruses (*Odobenus rosmarus*) [13], [49]. In marine mammals subambient pressure, or suction, is primarily generated by the rapid retraction and depression of the tongue via the hyolingual apparatus [20]–[23].

Pinnipeds are apex predators that comprise an important component of marine food webs. They exhibit an interesting range of feeding modes that include suction, biting, grip-and-tear, and filter feeding [50]–[53]. However, among pinnipeds only walruses, bearded seals, and leopard seals [13], [17], [48], [49] have been demonstrated to use suction, although indirect evidence suggests many more species use suction [51], [53]. Given the functional requirements of capturing prey underwater, and the dense and viscous physical properties of water, one would predict that the adaptations for prey acquisition would converge widely with other obligate underwater feeding vertebrates. That is, suction feeding should be a widespread feeding mode among pinnipeds and other marine mammals. Although phocids are thought to have a greater suction capability than otariids and otariids are presumed to predominantly use biting for capturing prey [51], there are few data to support or refute these claims. However, it is becoming clear that marine mammals use multiple feeding modes depending upon the need [17], [48]. Although recent data have demonstrated high performance values for suction feeding in bearded seals and walruses, these species are specialists among pinnipeds and may not represent the more generalized trophic ecology of many pinnipeds.

Among phocids, harbor seals (*Phoca vitulina*, Linnaeus, 1758) are the most widely distributed species globally and exhibit an opportunistic and generalized feeding ecology. Up to five subspecies are recognized: *P. v. vitulina* (Linneaus, 1858), *P. v. concolor* (DeKay, 1842), *P. v. mellonae* (Doutt, 1942), *P. v. richardii* (Gray, 1864), and *P. v. stejnegeri* (Allen, 1902) [54], [55], but there is much disparity in the morphological and genetic data and many of these subspecies are unsupported, or require further assessment. Harbor seals are generalists that feed upon a wide diversity of small- to medium-size fishes (e.g., herring, anchovy, cod, hake, trout, smelt, shad, scorpionfish, rockfish, prickleback, greenling, sculpin, capelin, sandlance, salmon, and flatfish), and a variety cephalopods, and invertebrates (mostly crab and shrimp species but also mollusks) [56]–[62]. Foraging can occur adjacent to haulouts, along rivers, or ∼50 km offshore from haulouts [63]. They can forage for food at considerable depths (∼500 m) or in shallow water [55], [64]. Diets vary regionally and seasonally, and prey availability is likely the driving force behind diet composition, however, the diet of different age classes also varies [65]. Despite the reported diversity of diet, often a few items will make up the bulk of seasonal diets, which vary with locality [65]. Some evidence suggests that harbor seals use several feeding modes including suction and biting [37]. Therefore, harbor seals are ideal candidates to test hypotheses regarding feeding performance of secondarily aquatic mammals that have implications for both proximal and ultimate questions. The objectives of this study were to investigate the feeding performance of harbor seals to begin to determine the range of their behavioral repertoire for capturing prey, and to test the hypothesis that suction feeding is their primary feeding mode, as opposed to biting. In addition, the hypothesis that rapid jaw opening, in addition to rapid tongue and hyoid depression, is correlated with suction feeding was tested. Since suction appears to be an important feeding mode, subambient (suction) and suprambient pressure (hydraulic jetting) generated by harbor seals were directly measured to test the hypothesis that harbor seal pressure generating capability is similar to values reported for bearded seals [17].

## Materials and Methods

### Subjects

This study was conducted at the Cologne Zoo (Cologne, Germany) and at the Marine Science Center at the University of Rostock (Rostock, Germany). Eight adult male harbor seals participated in this study (see Table 1 for details regarding subjects); the seals were well trained and were eager to participate in these novel tasks. All work was approved by Texas A&M University’s Institute of Animal Care and Use Committee Animal Use Protocol # 2010–67, and was conducted in accordance with the European Communities Council Directive of 24 November 1986 (86/609/EEC). All work at the Marine Science Center at the Cologne Zoo and at the University of Rostock was approved by both institutes.

**Table 1:** Experimental Subjects.

Subject	Birth year	Age (years)	Body Length (cm)	Mass (kg)	Birth Location
Filou	2006	4	130	68.4	captivity
Luca	2002	8	143	84.5	captivity
Malte	1999	11	141*	108.5	captivity
Nick	1999	11	161	102	captivity
Bill	1998	12	150	94	captivity
Henry	1997	13	151	75	captivity
Sam	1994	16	153	89	captivity
Marco	1982	28	143	85.5	unknown

Body length follows American Society of Mammalogists standards [77].

* This subject lacks the small tail in between the hind flippers (average tail length: 8 cm).

### Feeding Platform

**Figure 1:**
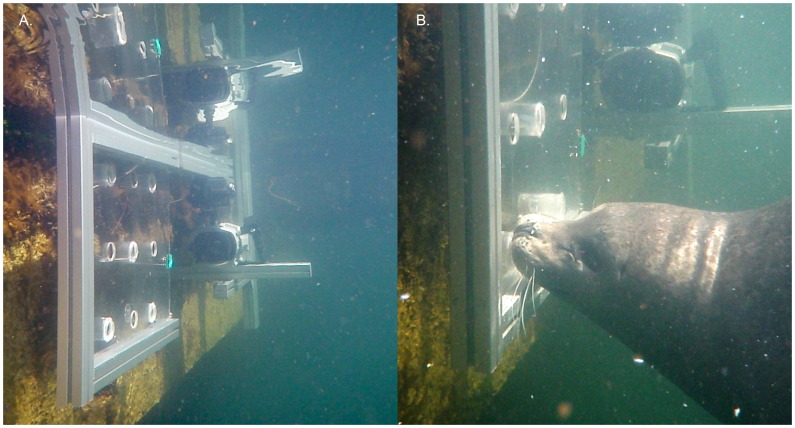
Experimental Feeding Platform. A. Feeding platform in place in the enclosure. B. Harbor seal feeding from feeding apparatus.

Feeding apparatuses were constructed to present subjects with food items (cut herring and sprat) in a controlled and repeatable research design. A plexiglass feeding panel was inserted vertically into the feeding apparatus with the feeding surface parallel to an underwater camera’s perspective (Fig. 1). Nine holes, 3.3 cm in diameter, were drilled through the plexiglass in three rows and three columns, 2 cm apart. Pieces of fish were presented to the subjects in two ways, simultaneously. Cut fish were pushed through the holes such that portions of fish projected out of the feeding surface, and were accessible to the subjects. In addition, fish were cut to fit within recessed plexiglass cylinders (inner diameter = 3.8 cm; length = 5.7 cm) that were positioned behind five of the holes through the plexiglass. Holes (1 cm diameter) were drilled through the back of each plexiglass cylinder to allow water flow. The feeding apparatus was placed in the water, suspended just below the surface, in the vertical plane (see Fig. 1). In addition, feeding trials were also conducted with the feeding apparatus out of the water. Subjects hauled out pool-side and were allowed to feed from the apparatus in the same manner as during the in-water trials.

By presenting food items projecting from holes in the feeding surface and within recessed cylinders during feeding trials, subjects were forced to make several choices when presented with food. Upon encountering food items, their first choice was whether to consume fish projecting from the holes or fish residing within the recessed cylinder. If seals chose to consume fish projecting from holes in the plexiglass feeding surface, then they had to choose whether to consume the piece of fish by biting and removing the fish with their teeth, or to use suction. If a subject chose to consume a piece of fish in the recessed cylinder, then its only option was to use suction to obtain that food item. In this way it was possible to determine whether subjects used biting or suction as their primary feeding mode. To challenge subjects, numerous pieces of fish were packed into recessed cylinders to elicit maximum suction generation. We used both kinematic and pressure data to categorize each feeding event as suction or biting. A hermetically sealed pressure catheter was placed through one of the cylinders to directly measure suction forces simultaneously with kinematic events (see below).

### Feeding Events, Kinematic Variables and Analyses

One hundred forty-four feeding events were recorded from eight subjects. From this dataset, ninety-one feeding events (from fifty-one feeding trials) from five subjects met the criteria for kinematic analyses. To meet our kinematic criteria, the cranial landmarks of each subject and food items had to be visible within each video frame during an entire feeding event, and rotation of the body around the longitudinal axis had to be minimal (<10°). Harbor seals had a propensity to roll as they fed but could successfully feed in any orientation. This necessitated conducting many more feeding trials than were ultimately used in the kinematic analyses. The number of feeding events used in the kinematic analyses was nearly equal between in-water and out-of-water trials (44 in-water vs. 47 out-of-water events). Subjects were videotaped at 60 Hz using a Sony camcorder within an underwater video housing (Fig. 1). Prior to feeding trials, zinc oxide landmarks were placed on the subject’s lips, jaws, and head to provide high contrast landmarks for digitizing. Homologous high-contrast landmarks were digitized frame-by-frame for motion analysis using Motus 9.0 motion analysis software system (Vicon, Denver, CO, USA). Digitized points were placed within spatial models and used to calculate kinematic variables.

The kinematic variables listed below were selected to determine the behavioral repertoire of prey capture, characterize the feeding mode of harbor seals, test the hypothesis that rapid jaw opening contributes to subambient pressure generation, and build upon our comparative feeding performance dataset for marine mammals [17]–[19]. Kinematic variables measured were: (1) maximum gape, the maximum distance from maxillary tip to mandibular tip; (2) time to maximum gape, the duration from when the lower jaw began to open until maximum gape; (3) maximum gape angle, the maximum angle from the maxillary tip to corner of the mouth to mandibular tip; (4) time to maximum gape angle; the duration from when the lower jaw began to open until maximum gape angle; (5) maximum opening gape angle velocity, the greatest angular rate of lower jaw opening; (6) time to maximal opening gape angle velocity, the duration from when the lower jaw began to open until maximum gape angle velocity was achieved; (7) maximum closing gape angle velocity, the greatest angular velocity during lower jaw closure; (8) time to maximum closing gape angle velocity, the duration from when the lower jaw began to close until maximum gape angle velocity was achieved; (9) maximum gular depression, the greatest increase in distance from the eye to external rostral border of the hyoid; and (10) time to maximum gular depression, the duration from start of gular depression to maximum gular depression. Total feeding cycle duration was also calculated.

Maximum gape and gape angle during feeding events was compared to the mean maximum biological gape and gape angle for all animals in the study. Each subject was digitally photographed while opening their mouth to their widest extent at the command of a trainer. Maximal biological gape and gape angle was measured from scaled digital photographs using ImageJ (National Institutes of Health, Bethesda, MD, USA).

### Pressure Measurements

A total of 667 pressure measurements from 144 feeding events and eight subjects were recorded simultaneously with kinematic events. Whereas numerous kinematic feeding events from several individual subjects did not meet the criteria for inclusion of kinematic analyses, many more of the pressure measurements did meet the criteria for inclusion for pressure analyses. Therefore many more pressure measurements were collected than kinematic feeding events. Pressure measurements were collected using a Millar MPC-500 catheter pressure transducer connected to a transducer control box (Millar TCB-600; Houston, TX, USA) and a Biopac MP150 portable electrophysiological recording system (Biopac, Oleta, CA, USA). Pressure data were saved to a laptop using Acknowledge software (Biopac, Oleta, CA, USA). To synchronize kinematics with pressure data, we used an electronic device that generated a square wave pattern and corresponding flashing pattern of dual LED lights. The LED display was affixed to the feeding platform and recorded by the camcorder. The square wave pattern was recorded as a second channel simultaneously with pressure data collection in Acknowledge. This allowed synchronization of the suction feeding behavior with pressure measurements in Acknowledge. The pressure transducer was calibrated using the control box, but also in the laboratory under a range of known pressure regimes. Prior to each feeding trial a pressure transducer was placed through the back of a recessed cylinder (that contained food) so that the tip of the transducer, where the recording element was located, projected ∼1 cm beyond the plexiglass feeding surface. This allowed the pressure sensor to be just at the seal’s lips, or slightly within the oral cavity, during feeding. This distance was verified visually during the feeding trials and from video footage. Only suction force data in which the tip of the transducer was at this location were analyzed. In addition, only pressure measurements that exceeded ±0.1 V (∼5 kPa) in magnitude were included in the data analysis. The maximum amplitude and duration of every subambient and suprambient pressure event was measured. Prior work on bearded seal pressure generation [23] demonstrated that suction generated when feeding from the apparatus did not differ from suction values collected during hand feeding.

### Statistics

Normality of data was tested using a Kolmogorov-Smirnov test. If normality was not met, then the data were log_10_ transformed. Levene’s test was used to test the assumption of homogeneity of variances. When both variance and normality requirements were met, one-way analyses of variance (ANOVAs) were performed to determine significant differences (*α*≤0.05) of kinematic variables and pressure data during feeding trials for the categorical treatment of biting vs. suction behaviors averaged from all five subjects that met the criteria for inclusion in the statistical analyses. A Principal Component (PC) analysis on correlations was performed on the kinematic data as a tool to explore the correlation of kinematic variables. A PC analysis on correlations was used instead of PC on covariances since our variables have different units of scale, and a PC analysis on the correlation matrix is a way of standardizing such variables. Pearson’s correlation analysis assessed the positive or negative correlation of the timing and displacement variables of feeding events. All statistical tests were conducted using JMP 9.0 (SAS Institute, Cary, North Carolina, USA).

## Results

### Biting vs. Suction Behavior

While all subjects used suction and biting when feeding underwater, suction was favored extensively (84% of all feeding events) compared to biting (16%). However, biting was still an important feeding mode. Food items that projected from a hole (i.e., not within a recessed cylinder) were usually consumed first using suction, but food items were also removed by grasping the food with the mouth and teeth (biting), and removing the fish from the apparatus. Food items placed within a recessed cylinder were removed using suction. These food items were more difficult to acquire and required more effort to consume. On a few occasions subjects were able to consume a recessed piece of fish by grasping and pulling on a small piece of fin with their incisors, just enough to place pursed lips on it, and remove the food using suction. Fish placed within the recessed cylinders also initiated hydraulic jetting behavior, which was often alternated with suction generation, but could also be used independently. Since this behavior is related to suction it is not consider to be one of the four feeding modes often described [30]. Hydraulic jetting was evident by water flow and turbulence appearing at the back of the recessed wells. When only a single piece of fish was left in a cylinder, alternation of suction with hydraulic jetting caused the food item to oscillate back-and-forth within the recessed cylinder until it came free and was consumed. After consuming fish, subjects routinely expelled water from the corner of their mouths after a suction event, which was evident from water turbulence emanating from the caudal lips and mouth corner, as well as the bulging out of the lips and soft tissue in this region. In general, subjects became proficient at consuming all fish pieces, regardless if they were recessed or not.

Suction feeding could be clearly distinguished from biting by the reduced gape from casual observation and by kinematic analyses (see below). Suction feeding was characterized by pursing of the lips to form a circular aperture, sealing of the lips to occlude lateral gape, and a cranial-to-caudal wave of motion in the gular region indicating hyolingual depression. A biting feeding mode was characterized by increased gape, lip curling with exposed teeth, and the lack of marked gular depression. The recessed cylinders of the feeding apparatus were designed to make food extraction difficult so that maximum suction and hydraulic jetting performance could be measured. Holes in the plexiglass were slightly narrower than the inner diameter of the plexiglass cylinder, which created a ridge (0.7 to 1 cm) between the cylinder and the location where seals placed their muzzle. To extract fish, enough pressure had to be employed to pull the food item up and over this ridge. In addition, numerous pieces of fish were packed into each cylinder in an effort to make extraction difficult and elicit powerful suction forces. The alternating use of suction with hydraulic jetting was successful because movement of the food items increased the chance that the food item could be lifted over the ridge. The appearance of bubbles from turbulence flowing from the back of the recessed cylinders toward the subject’s lips during suction events, alternating with cloudy plumes of minute fish particles and scales exiting the back of the recessed wells during hydraulic jetting events, in addition to direct pressure measurements (see below), was further evidence that suction and hydraulic jetting were being used. Feeding trials on-land elicited only biting behavior (100%). Biting events on land involved subjects grasping projecting fish items with their teeth, removing them from the apparatus and ingesting the food. Occasionally fish were bit in half and consumed, leaving pieces of fish in the apparatus.

### Vibrissal Use

Mystacial vibrissae were used during all feeding trials regardless of location. During on-land feeding trials, it appeared that subjects used vision in addition to other senses since the eyes often remained open. During in-water feeding trials, subjects always fed with their eyes closed and appeared to rely more on active touch sensation using mystacial vibrissae. Seals were observed to use the largest of the mystacial vibrissae to explore the edges of the feeding apparatus and to locate pieces of fish protruding from holes in the plexiglass surface. However, for food held within recessed cylinders, subjects would systematically locate the center of each cylinder by sweeping the plexiglass surface with their most medially located vibrissae and allow these whiskers to protrude into each cylinder. If a food item was still located within a cylinder, these whiskers could often touch a food item. This would elicit suction behavior and if the food item was difficult to remove it would then elicit hydraulic jetting behaviors, often alternating with suction behavior, until the food items(s) were removed and consumed.

### Feeding Kinematics

As characterized in bearded seals [23], four feeding phases were differentiated: (I) preparatory, (II) jaw opening, (III) gular depression, and (IV) jaw closing were observed regardless of the feeding mode. Phase I began at the onset of jaw opening and ended when gape increased by greater than 0.2 cm field^−1^ (1 field = 60 Hz) and the jaws rapidly opened. Phase II began when gape increased by ≥0.2 cm field^−1^ and persisted until maximum gape. Phase III began when gular depression increased by ≥0.2 cm field^−1^. This phase overlapped with Phases II and IV, persisted the longest in duration, and concluded when gular depression returned to its original position, which was often at the end of the feeding event. Phase IV began at maximum gape and concluded when the jaws closed. The timing of maximum gular depression during suction feeding events always followed maximum gape or coincided with maximum gape. Little to no gular depression was observed during biting feeding events, whether seals were feeding in-water or on-land. The mean durations for Phases I–IV were, 0.02±0.001 s, 0.08±0.04 s, 0.16±0.10 s, and 0.11±0.07 s, respectively.

**Figure 2:**
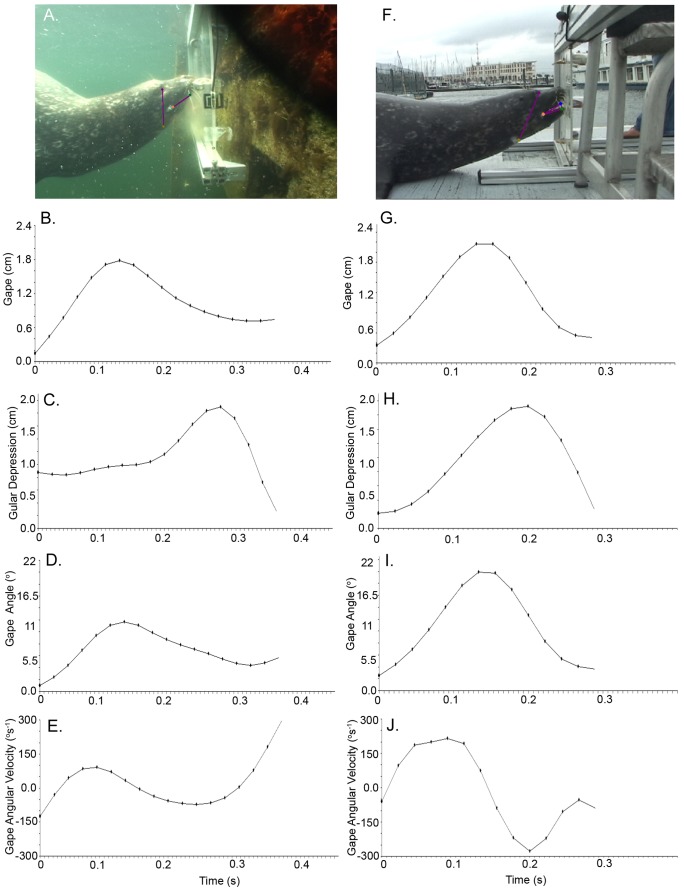
Representative Kinematic Profiles of Suction vs. Biting. A. Frame from video during in-water suction feeding trial with overlaid spatial model stick figure. B. Plot of Gape (cm) for a single suction feeding trial. C. Plot of Gular Depression (cm) for a single suction feeding trial. D. Plot of Maximum Gape Angle (degrees) for a single suction feeding trial. E. Plot of Gape Angle Velocity (degrees/s; opening and closing) for a single suction feeding trial. F. Frame from video during on-land biting feeding trial with overlaid spatial model stick figure. G. Plot of Gape (cm) for a single biting feeding trial. H. Plot of Maximum Gape Angle (degrees) for a single biting feeding trial. I. Plot of Gular Depression (cm) for a single biting feeding trial. J. Plot of Gape Angle Velocity (degrees/s; opening and closing) for a single biting feeding trial.

The mean feeding cycle duration for suction events was significantly shorter (0.15±0.09 s S.E.; P<0.01) than the mean feeding cycle duration during biting feeding events (0.18±0.08 s; Fig. 2, Table 2). This reflected divergent kinematic profiles between suction and biting feeding events. Suction was characterized by a significantly smaller gape (1.3±0.23 cm; P<0.001) and gape angle (12.9±2.02°; P<0.001) compared to biting events, which was characterized by a large gape (3.630±0.21 cm) and gape angle (28.8±1.80°; P<0.001). Gape and gape angle during suction or biting events was significantly less than maximum biological gape and gape angle (P<0.001). Mean maximum biological gape and gape angle from all seals that participated in the study was 10.1±0.80 cm and 69.7±2.66°, respectively. Time to maximum gape and gape angle were significantly shorter during suction events (0.07±0.01 s each respectively; P<0.01 and P<0.02, respectively) compared to biting events (0.08±0.01 s each respectively). Maximal gape angle opening velocity (GAOV) was significantly slower during suction events (197.2±42.04°s^−1^; P<0.001) than during biting events (561.6±37.61°s^−1^). However, time to GAOV for suction (0.05±0.01) vs. biting (0.04±0.01) events was not significantly different. Maximal gape closing angle velocity (GACV) was significantly slower during suction events (159.1±42.05°s^−1^; P<0.001) than biting events (544.2±37.61°s^−1^); time to GACV was significantly longer for suction (0.11±0.01 s; P<0.001) compared to biting events (0.13±0.01). Gular depression was significantly greater, and time to maximum gular depression was significantly longer, during suction feeding events (1.4±0.08 cm, 0.14±0.01 s, respectively; P<0.001) compared to biting events (0.9±0.07 cm, 0.07±0.01 s, respectively). Figure 2 depicts representative kinematic profiles for suction and biting; kinematic variables of biting and suction feeding are summarized in Table 2.

**Table 2:** Summary of Kinematic Variables.

Kinematic Variable	Suction	Biting	P
Max. Gape (cm)	1.3±0.23	3.6±0.21	0.001
Time to Max. Gape (s)	0.07±0.01	0.08±0.04	0.01
Maximum Gape Angle (^o^)	12.9±2.02	28.2±1.8	0.001
Time to Max. Gape Angle (s)	0.07±0.01	0.08±0.04	0.02
Max. GAOV (deg. s^−1^)	197.2±42.04	561±37.61	0.001
Time to Max. GAOV (s)	0.05±0.01	0.04±0.01	0.172
Max. GACV (deg. s^−1^)	159.1±42.05	544.2±37.61	0.001
Time to GACV (s)	0.11±0.01	0.13±0.01	0.001
Max. Gular Depression (cm)	1.4±0.08	0.9±0.07	0.001
Time to Max. Gular Depression (s)	0.14±0.01	0.07±0.01	0.001
Feeding Cycle Duration (s)	0.15±0.09	0.18±0.08	0.01

Values are means ± S.D., N = 5, 91 feeding events, GAOV = Gape Open Angle Velocity, GACV = Gape Angle Close Velocity.

Principal component (PC) analysis on correlations of log-transformed data demonstrated that the first 3 PC axes characterized 78% of the variation of harbor seal feeding kinematics (PC1 = 49.986%, PC2 = 18.949%, PC3 = 9.072%; Table 3). High loadings on PC axis 1 identified most kinematic variables, with the exception of maximum gular depression and time to maximum gular depression. PC axis 2 identified all timing kinematic variables with the exception of time to maximum gape angle closing velocity. In addition PC axis 2 had a high loadings\ for maximum gular depression. PC axis 3 had high loadings for only maximum gular depression and time to maximum gular depression, indicating distinct differences in suction vs. biting feeding events. Pearson’s correlation analysis further supported the difference between suction and biting feeding kinematics and is summarized in Table 4. As shown by the PC analysis, the Pearson’s correlation analysis demonstrated that most, but not all, kinematic variables were positively correlated. However, gular depression and time to gular depression were distinct in that they were negatively correlated with most kinematic variables.

**Table 3:** Loadings for Principal Components Axes 1–3.

Kinematic Variable	PC 1	PC 2	PC 3
Max. Gape (cm)	0.87150	−0.26014	0.21490
Time to Max. Gape (s)	0.74441	0.45444	−0.17919
Maximum Gape Angle (^o^)	0.89261	−0.26282	0.25726
Time to Max. Gape Angle (s)	0.73908	0.44899	−0.17911
Max. GAOV (deg. s^−1^)	0.82027	−0.31931	−0.27337
Time to Max. GAOV (s)	*0.38675*	0.64345	−0.17341
Max. GACV (deg. s^−1^)	0.82532	−0.34010	0.21580
Time to GACV (s)	0.75757	0.27698	−0.20563
Max. Gular Depression (cm)	−0.24685	0.53656	0.70944
Time to Max. Gular Depression (s)	−0.36336	0.66035	0.37378
Feeding Cycle Duration (s)	0.75588	0.32745	−0.11137

Log_10_ transformed data, N = 5, 91 feeding events, GAOV = Gape Open Angle Velocity, GACV = Gape Angle Close Velocity.

**Table 4:** Pearson’s Correlation of Kinematic Variables.

	Max. Gape	Time toMax. Gape	Max. Gape Angle	Time to Max. Gape Angle	Max. GAOV	Time Max. GAOV	Max. GACV	Time Max. GACV	Max. Gular Depress	Time Max. Gular Depress	Feeding Cycle Duration
**Max. Gape**	1.0000	0.4684	0.8942	0.5192	0.7947	0.1201	0.8099	0.5620	−0.2153	−0.3877	0.5336
**Time to Max. Gape**	0.4684**	1.0000	0.4760	0.9460	0.4053	0.4791	0.4635	0.5501	−0.0281	−0.0694	0.6181
**Max. Gape Angle**	0.8942**	0.4760**	1.0000	0.4727	0.8782	0.1857	0.8785	0.5656	−0.2290	−0.3481	0.5340
**Time to Max. Gape Angle**	0.5192**	0.9460**	0.4727**	1.0000	0.4117	0.4986	0.4068	0.5264	−0.0191	−0.0937	0.5850
**Max. GAOV**	0.7947**	0.4053**	0.8782**	0.4117**	1.0000	0.1409	0.7774	0.4708	−0.1659	−0.4500	0.4634
**Time Max. GAOV**	0.1201	0.4791**	0.1857	0.4986**	0.1409	1.0000	0.0853	0.4346	0.1341	0.1276	0.4082
**Max. GACV**	0.8099**	0.4635**	0.8785**	0.4068**	0.7774**	0.0853	1.0000	0.4734	−0.2627	−0.3992	0.4468
**Time Max. GACV**	0.5620**	0.5501**	0.5656**	0.5264**	0.4708**	0.4346**	0.4734**	1.0000	−0.1696	−0.0494	0.8069
**Max. Gular Depression**	−0.2153*	−0.0281	−0.2290*	−0.0191	−0.1659	0.1341	−0.2627*	−0.1696	1.0000	0.5079	−0.0917
**Time Max. Gular Depress.**	−0.3877**	−0.0694	−0.3481**	−0.0937	−0.4500**	0.1276	−0.399**	−0.0494	0.5079**	1.0000	−0.0702
**Feeding Cycle Duration**	0.5336**	0.6181**	0.5340**	0.5850**	0.4634**	0.4082**	0.4468**	0.8069**	−0.0917	−0.0702	1.0000

Log_10_ transformed data, GAOV = Gape Open Angle Velocity, GACV = Gape Angle Close Velocity, * = P<0.05, ** =  P<0.01, Bold = positive correlation, Italics = negative correlation, N = 5, 91 feeding events.

### Suction and Hydraulic Jetting Pressures

**Figure 3:**
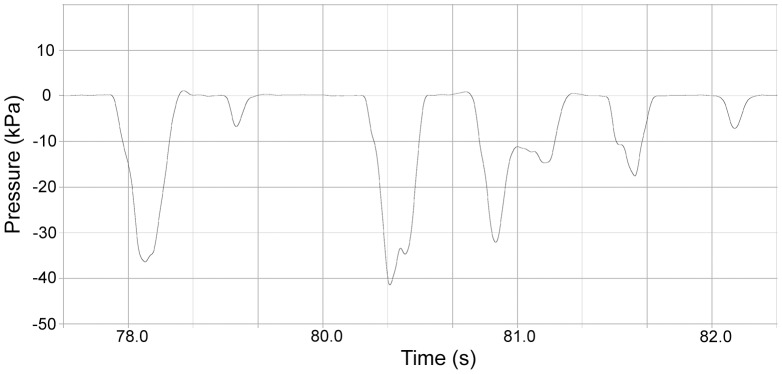
Pressure Traces of Subambient Pressure Data. A series of six suction events of varying magnitude.

**Figure 4:**
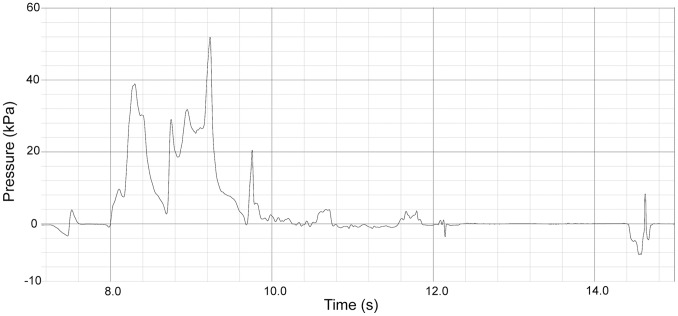
Pressure Traces of Suprambient Pressure Data. A series of seven hydraulic jetting events.

Direct measurement of sub- and suprambient pressure generation during feeding events supported the observational data that harbor seals primarily used suction (subambient pressure generation) when feeding in-water. However, hydraulic jetting (suprambient pressure generation) was also employed. Frequency data from pressure traces show that subjects in this study employed suction 90.5% of the time and hydraulic jetting 9.5% of the time. Pressure data demonstrate that suction events were composed of an expansive phase, during which the maximum subambient pressure was reached for that event, followed by a compressive phase, during which pressure returned to baseline levels (Fig. 3). A preparatory phase was not observed. Similarly, pressure data from hydraulic jetting (Fig. 4) demonstrated only an expansive phase during which maximum suprambient pressure was recorded, followed by a compressive phase, during which pressure values returned to baseline. As with subambient pressure traces, a preparatory phase was never observed. The maximum subambient pressure recorded was 48.6 kPa and the maximum suprambient pressure recorded was 53.9 kPa. The mean duration of subambient pressure events (0.5±0.3 ms) was significantly longer (P<0.001) than the mean duration of suprambient pressure events (0.35±0.25 ms).

## Discussion

This study provides the first detailed kinematic and physiological data of feeding performance in harbor seals. The data support our hypothesis that suction is the primary underwater feeding mode in harbor seals. However, biting is also an important feeding mode, and likely more important than for suction feeding specialists such as bearded seals and walruses. The primary finding of this study was that suction was used predominantly underwater, but subjects were also capable of using hydraulic jetting either alone or in conjunction with suction feeding. The combined use of suction and hydraulic jetting for feeding has been documented previously for bearded seals [17], walruses [13], [49] and leopard seals [48]. Walruses excavate bivalves using hydraulic jetting to clear the sediment and then employ suction to remove all or part of the bivalve [13], [66]. The use of suction and hydraulic jetting have also been observed in cetacean suction feeding specialists such as pygmy and dwarf sperm whales [19], belugas [18] and pilot whales [15], [18]. All of these species, including harbor seals (this study), used hydraulic jetting either alone or alternating with suction feeding. The behavior of hydraulic jetting is likely similar to that of suction generation, but where the tongue and hyoid are elevated, instead of retracted, to produce suprambient pressures rather than subambient pressures. Although similar behaviorally, the underlying mechanisms (i.e., muscles of tongue retraction vs. protraction) may differ. However whether this is manifested in sub- vs suprambient pressure performance differences currently remains unclear. In this study, and in others [17], [18], maximum subambient and maximum suprambient pressures were similar in magnitude (48.6 kPa and 53.9 kPa, respectively). Harbor seals demonstrated several feeding mechanisms and modes that were used to capture prey.

The hypothesis that rapid jaw opening is correlated with suction feeding in harbor seals was not strongly supported in this study. Although harbor seals employed several of the same mechanisms to generate subambient pressure that are employed by other suction feeding specialists (closure of lateral gape, pursing of lips to form a circular aperture, and rapid hyolingual depression [13], [17], [48], [49], they did not employ a preparatory phase for suction that likely increases the change in intraoral volume that is related to greater subambient pressure development as reported for bearded seals [17]. This is likely related to the lack of a vaulted palate, observed in other pinnipeds specialized for suction [13], [17], [51], [53], [55] in harbor seals. Nor was rapid jaw depression (as measured by GAOV), which can also contribute to subambient pressure generation via further buccal expansion in conjunction with hyolingual depression, high compared to other marine mammal suction feeders, such as pygmy sperm whales (GAOV = 293 deg s^–1^) [19] and bearded seals (GAOV = 205 deg s^–1^) [17]. However, it is becoming apparent that the underlying mechanism of suction feeding is not homogeneous among all species for which data are available, and not all suction feeding specialists employ rapid jaw depression (i.e., belugas, GAOV = 119°s^−1^ [18]). As a result, the data in this study show that although harbor seals can create significant subambient pressures, their suction and hydraulic jetting performance are not at the same level for pinniped suction feeding specialists such as bearded seals (91.2 kPA [13]) and walruses (91.2 kPa [25] and 118 kPa [49]).

Harbor seals in this study used the large lateral mystacial vibrissae for exploration of the large scale features of the feeding apparatus, but then shifted to using the small medially located mystacial vibrissae for more refined and discrete tactile exploration. In particular, these small medially located mystacial vibrissae were used to locate the center of each cylinder and to protrude into each cylinder to touch recessed food items, if possible. Our observation that harbor seals use different regions of the mystacial vibrissae during feeding supports the results of more focused active touch performance studies [67], [68] in which harbor seals used the smaller medial mystacial vibrissae for detailed size discrimination, but within a different context. This active touch exploratory pattern is likely a typical pattern of how harbor seals explore new objects in their environment. Such exploratory behavior and use of different regions and size of mystacial vibrissae has also been observed in California sea lions [69], manatees and dugongs [45], [47], walruses [70], and rodents [71]–[73] and likely represents a generalized mammalian pattern of tactile exploration.

Data from this study show that harbor seals have a wide repertoire of feeding strategies that include biting, suction, and hydraulic jetting. Behavioral observations also showed that harbor seals were flexible and creative in extracting food items from the feeding apparatus. Biting in-water appeared to involve smaller gapes than biting on–land, but this currently remains unclear and needs to be investigated further. Such flexibility of feeding strategies and biomechanics likely form the basis of their opportunistic, generalized feeding ecology and concomitant breadth of diet. Although there were many similarities in the kinematic profile of harbor seals compared to bearded seals, harbor seals lacked certain behaviors (i.e., lack of preparatory phase prior to suction feeding and slower jaw depression) that could have increased their subambient pressure generation. However, the subambient pressures produced were still significant and are likely strong enough to be an important feeding mode for harbor seals. In addition, like bearded seals, it is probable that harbor seals can use the substrate and the geometry of the habitat to passively increase their suction, and possibly their hydraulic jetting, capability. The positive effect of the substrate to passively increase the suction capability of foragers has been documented in both chondrichthyan and teleost fishes [74]–[76] and is the result of the conservation of momentum of water flow [76]. This strategy likely has important trophic implications, such as increasing prey-capture efficiency, for any suction feeding marine mammal, as it does for benthic foraging fishes.
